# RIPK2: a promising target for cancer treatment

**DOI:** 10.3389/fphar.2023.1192970

**Published:** 2023-05-30

**Authors:** Jieqiong You, Ying Wang, Haifeng Chen, Fang Jin

**Affiliations:** ^1^ Shanghai Frontier Health Pharmaceutical Technology Co. Ltd, Shanghai, China; ^2^ Shanghai Linnova Pharmaceuticals Co. Ltd, Shanghai, China; ^3^ State Key Laboratory of Microbial Metabolism, Department of Bioinformatics and Biostatistics, National Experimental Teaching Center for Life Sciences and Biotechnology, School of Life Sciences and Biotechnology, Shanghai Jiao Tong University, Shanghai, China

**Keywords:** RIPK2, tumor, targeted therapy, RIPK2 kinase inhibitors, PROTACs

## Abstract

As an essential mediator of inflammation and innate immunity, the receptor-interacting serine/threonine-protein kinase-2 (RIPK2) is responsible for transducing signaling downstream of the intracellular peptidoglycan sensors nucleotide oligomerization domain (NOD)-like receptors 1 and 2 (NOD1/2), which will further activate nuclear factor kappa-B (NF-κB) and mitogen-activated protein kinase (MAPK) pathways, leading to the transcription activation of pro-inflammatory cytokines and productive inflammatory response. Thus, the NOD2-RIPK2 signaling pathway has attracted extensive attention due to its significant role in numerous autoimmune diseases, making pharmacologic RIPK2 inhibition a promising strategy, but little is known about its role outside the immune system. Recently, RIPK2 has been related to tumorigenesis and malignant progression for which there is an urgent need for targeted therapies. Herein, we would like to evaluate the feasibility of RIPK2 being the anti-tumor drug target and summarize the research progress of RIPK2 inhibitors. More importantly, following the above contents, we will analyze the possibility of applying small molecule RIPK2 inhibitors to anti-tumor therapy.

## Introduction

Chronic inflammatory diseases like inflammatory bowel disease (IBD), rheumatoid arthritis (RA), and psoriasis comprise a group of disorders in which deregulation of the immune systems plays a pivotal role in establishing and maintaining disease (Ozkurede and Franchi, 2012; Broderick et al., 2015; Bertani, 2022). Common to these diseases is an excessive inflammatory response, causing the production and release of inflammatory cytokines and chemokines that accelerate a vicious cycle of inflammation with the immune system unable to resolve this cascade. This abnormal inflammatory response results in tissue destruction and impaired mucosal healing of the gastrointestinal tract in IBD, tissue destruction in joints accompanied by joint pain and swelling in RA, and dermatosis in psoriasis (Podolsky, 2002; Xavier and Podolsky, 2007; Armstrong and Read, 2020; Tartey and Kanneganti, 2020).

Cumulative evidence has shown that aberrant activation of innate immune signaling is involved in the occurrence and development of autoimmune diseases, and nucleotide-binding oligomerization domain-like receptor (NLR), a family of evolutionarily conserved innate immune receptors, plays essential roles in various autoimmune diseases (Cooney et al., 2010). NLRs consisting of a C-terminal leucine-rich repeat (LRR), a central nucleotide-binding domain, and an N-terminal effector domain, form a group of pattern recognition receptors (PRRs) that mediate the immune response by specifically recognizing cellular pathogen-associated molecular patterns (PAMPs) or damage-associated molecular patterns (DAMPs) and triggering numerous signaling pathways, including RIPK2 kinase, caspase-1, NF-κB, MAPK and so on (Ogura et al., 2001; Philpott et al., 2014; Li and Wu, 2021).

Notably, the NOD2-RIPK2 pathway has attracted particular attention due to its role in granulomatous inflammatory diseases, including IBD (Gutierrez et al., 2002). RIPK2 signaling relies on the N-terminal kinase domain with dual Ser/Thr and Tyr kinase activities as well as the C-terminal caspase activation and recruitment domain (CARD), which mediates the assembly of the CARD-CARD domain with activated NODs (Gong et al., 2018). Once engaged, RIPK2 is firstly activated by autophosphorylation and further targeted by X-linked inhibitor of apoptosis (XIAP) and other E3 ligases for non-degradative polyubiquitination, such as the linear ubiquitin chain assembly complex (LUBAC). With the recruitment of TGF-Beta activated kinase 1 binding protein 1 (TAB1) and TAB2/3, ubiquitin-coupled proteins subsequently activate TGF-Beta activated kinase 1 (TAK1) and result in the activation of MAPK/c-Jun N-terminal kinase (JNK)/p38/extracellular regulated protein kinases (ERK) kinase signaling pathways and NF-κB (Kobayashi et al., 2002; Adhikari et al., 2007; Krieg et al., 2009; Gong et al., 2018; Topal and Gyrd-Hansen, 2021).

Except for autoimmune diseases, pathologies like tumors can be caused by the dysregulation of the immune system. So far it is been clear that the tumor microenvironment, which is largely orchestrated by chronic irritation and inflammation, is a necessary participant in the neoplastic process, fostering proliferation, survival, and invasion (Coussens and Werb, 2002). During the development of chronic inflammation, the NF-κB pathway has long been considered as a prototypical proinflammatory signaling pathway, which is hyperactivated at high frequencies in the tumor (Taniguchi and Karin, 2018). Therefore, it comes as no surprise to infer that one of the main NF-κB regulators, RIPK2, is also highly expressed and unfriendly to antitumor therapy. Data from GEPIA 2 (http://gepia2. cancer-pku.cn/#general) have exactly illustrated that RIPK2 predominantly expresses in the human breast, skin, and lung tissues plus blood system, and is upregulated in various types of tumors, such as breast, ovarian, colon, esophagus, stomach, and pancreas cancers (Tang et al., 2019). As the noteworthy association between RIPK2 expression level and tumorigenesis has been established, RIPK2 should be regarded as a potential target for cancer therapeutic intervention. In this review, we will give an overview of the integrated role of RIPK2 in the progression of tumor malignancy and the feasibility of RIPK2 as an anti-tumor therapeutic target.

## Manuscript

### RIPK2 promotes the malignant progression of cancer

#### Gynecological tumors

##### Breast cancer (BRCA)

The amplification of ErbB2 (HER2) occurs in approximately 10%–34% of BRCA, which is a predictor of high credibility against BRCA recurrence and survival. The growing drug resistance concern towards human epithelial growth factor receptor-2 (HER2)-targeted therapy subsequently spawned a new goal in breast cancer research, to be specific, the identification of druggable kinases beyond HER2. According to the research of proteogenomic analysis of The Cancer Genome Atlas (TCGA) samples, RIPK2 is highly expressed and has a high amplification rate in BRCA, which exhibited similar gene amplification-driven proteogenomic patterns to HER2(Mertins et al., 2016). Another study about pan-cancer analysis of the carcinogenic role of RIPK2 also illustrated that the gene amplification rate of RIPK2 in BRCA and uterine carcinosarcoma (UCS) was approximately 8.5% (Zhang et al., 2022a). Therefore, RIPK2 is likely to become a convincing predictor of BRCA recurrence and survival in the future, on the other hand, inhibition of RIPK2 could be a reasonable choice for BRCA-targeted therapy, especially in HER2 negative status.

In the molecular classification of HER2-negative breast cancer, triple-negative breast cancer (TNBC) has the poorest prognosis than any other type of breast cancer. The expression of RIPK2 in TNBC is higher than that of other molecular subtypes, and its high expression is negatively related to the prognosis of TNBC. Mechanistically, RIPK2 directly contributes to the tumor’s multiplication, invasion, and metastasis by promoting NF-κB and JNK activation (Singel et al., 2014; Jaafar et al., 2018). Hence, these results not only highlight that RIPK2 is a novel prognostic biomarker in breast cancer, but also suggest that targeting RIPK2 may improve the outcome of advanced breast cancer patients with RIPK2 amplification or overexpression.

##### Ovarian cancer (OC)

Serous ovarian cancer is a type of epithelial ovarian cancer that is conventionally treated with surgery and chemotherapy based on platinum agents and paclitaxel (Karnezis et al., 2017; Freimund et al., 2018). However, paclitaxel resistance is one of the primary factors for the poor prognosis. In a bioinformatics study searching for potential biomarkers associated with paclitaxel resistance in OC treatment, researchers found a correlation between the higher expression of RIPK2 and the development of paclitaxel resistance (Shen et al., 2022). The mechanism leading to the resistance, for one thing, depends on the over-activation of RIPK2-mediated NF-κB signaling pathway, and another facet is the change of tumor microenvironment caused by RIPK2-mediated immune infiltration, including CD4^+^ memory T-cell, dendritic cells (DCs), common lymphoid progenitors (CLPs) (Shen et al., 2022; Zhang and Wang, 2022). Therefore, this makes sense to choose RIPK2 as a candidate target for OC, as RIPK2 inhibitors could be used in combination with chemotherapy agents to grapple with potential drug resistance and significantly ameliorate the original immune microenvironment that promotes tumor progression.

#### Gastrointestinal cancers

##### Colorectal cancer (CRC)

As an important mediator required for immune and inflammatory response, RIPK2 is closely related to the occurrence and development of IBD. Considering the chronic inflammatory intestine microenvironment of IBD patients and the essentiality of inflammation in the incidence of CRC, the susceptibility to CRC in IBD patients is likely to be conspicuously increased by comparison with the normal population (Stronati et al., 2010; Rubin et al., 2012; Axelrad et al., 2016). Moreover, the association between RIPK2 and vulnerability to CRC has been directly confirmed in a recent study of CRC patients. RIPK2 was found to be significantly upregulated in rectal tumor tissues compared with normal adjacent mucosa, suggesting that RIPK2 plays a vital role in the progression of IBD to CRC (Flebbe et al., 2019).

To understand how RIPK2 promotes the malignant progression of CRC, a correlation analysis between RIPK2 expression level and cytokines involved in the progression of CRC hinted that patients with high RIPK2 expression also had higher secretion levels of interleukin (IL)-6, IL-8, and vascular endothelial growth factor (VEGF). However, the increased secretion of these factors is not conducive to the improvement of prognosis and CRC patients’ survival rate (Jaafar et al., 2021). Accordingly, in the regulation network whose function can be interfered with, Mir-146a is found to be a negative regulator of RIPK2, which further exerts anti-tumor effects on CRC through RIPK2 inhibition and the following limitation on bone marrow-mediated inflammatory IL-17 production and IL-17 signaling transduction (Garo et al., 2021). From this, we can find firm evidence for RIPK2 inhibition in the treatment of inflammation-related cancers, i.e., CRC. Furthermore, results from the RIPK2-based regulatory network casts a new light on development strategies for RIPK2 inhibitors. The inhibition of RIPK2 should not be confined to direct inhibition, indirect interference with RIPK2 protein function through amplification of negative regulatory signals and interdiction of positive regulatory signals also has certain feasibility.

##### Hepatocellular carcinoma (HCC)

Chronic inflammation caused by excessive drinking, aflatoxin intake, or hepatitis B/C virus infection is the most important risk factor for developing liver cirrhosis, and finally HCC (Badvie, 2000; Matsuzaki et al., 2007). Notably, tumor necrosis factor (TNF)-α and IL-6 played pivotal roles in inflammation-induced HCC tumorigenesis and progression. In tracing the upstream regulators of TNF-α and IL-6, Pim-2 proto-oncogene, serine/threonine kinase (PIM2) was discovered as an upstream candidate gene (Fox et al., 2003; Asano et al., 2011). The following study further provides evidence of the oncogenic function of PIM2 in HCC. In particular, a feedback loop between PIM2 and TNF-α becomes the driving force from chronic liver inflammation to HCC. The expression level of PIM2 can be upregulated by the stimulation of TNF-α, and the abnormal expression of PIM2 in HCC cells can in turn promote the NF-κB mediated transcription of TNFα through the phosphorylation of RIPK2 (Tang et al., 2020).

Apart from the alteration of the tumor microenvironment, regulatory networks have also been discovered between RIPK2 and tumor driver genes. It is by now generally accepted that c-Myc amplification is the culprit that promotes malignant progression, particularly in liver cancer (Kawate et al., 1999; Shachaf et al., 2004; Cancer Genome Atlas Research Network. Electronic address and Cancer Genome Atlas Research, 2017). However, none of the c-Myc targeted agents have been approved by Food and Drug Administration (FDA) so far, which is mainly due to c-Myc being a non-enzymatic transcription factor as well as the potential off-target effects and subsequent toxicity that are difficult to estimate after inhibition (Huang et al., 2014; Chen et al., 2018a). According to the research from Yan *et al,* RIPK2 phosphorylates, stabilizes, and activates c-Myc through activating RIPK2/MKK7(/JNK)/c-Myc signaling axis, inhibition of RIPK2 by gene silence or small molecule inhibitors effectively blocks the phosphorylation cascades, resulting in instability of the c-Myc protein and metastasis inhibition (Yan et al., 2022). Besides, in tumor cell lines examined with high expression of c-Myc and PIM1/2 kinases, knockdown of Pim kinases significantly reduced endogenous c-Myc expression level, which caused the suppression of cell transformation and proliferation. This implies that the strong synergy between these two proto-oncogenes is associated with tumorigenesis (Zhang et al., 2008). More interestingly, RIPK2 may play a part in the c-Myc/Pim synergistic impact on malignant progression, as RIPK2 is one of the PIM2 substrates to be phosphorylated (Fox et al., 2003). Together with the sample analysis result that the activity score of RIPK2 is highly correlated with c-Myc activity scores in clinical tissue specimens of 32 cancer types (Yan et al., 2022), we speculate that RIPK2 could also be a potential target for HCC treatment with high reliability.

##### Others

In addition to colorectal and liver tumors, the abnormal activation of RIPK2 is also allied to the malignant progression of other digestive tumors, which consists of gastric cancer and esophageal squamous cell carcinoma (Montenegro et al., 2020; Yang et al., 2021; Nomoto et al., 2022). From the mechanistic point of view, the unrestrained proliferation, metastasis, and apoptosis inhibition are dependent on the NF-κB signaling pathway over-activated by RIPK2 (Yang et al., 2021; Nomoto et al., 2022). Taken together, a variation in RIPK2 expression level could serve as a new molecular marker for auxiliary diagnosis and molecular classification of tumors mentioned above. As for being the new target to shrink tumors, such expositions are unsatisfactory because the specificity and importance of the NF-κB signaling pathway in tumor pathogenesis are still not clearly defined.

#### Head-neck cancers

##### Oral squamous cell carcinoma (OSCC)

As far as we know, the mechanism of IL-8 promoting tumor progression is mainly through its role as an autocrine growth factor and angiogenesis factor, and the concentration of IL-8 in the saliva of OSCC patients is higher than that of normal cohorts, which indicates that IL-8 is a potential biomarker and intervention target for OSCC (Hwang et al., 2012; Lisa Cheng et al., 2014). In a correlation study of IL-8 and OSCC, IL-8 silence effectively harms to OSCC cell viability and colony formation since IL-8 works through C-X-C motif chemokine receptor 1 and 2 (CXCR1/2)-mediated NOD1/RIPK2 signaling pathway activation (Chan et al., 2016). That is to say, the dysfunction of NOD1 signaling pathways may be associated with OSCC progression, and both NOD1 and RIPK2 could be used as potential novel biomarkers for oral carcinogenesis (Wang et al., 2014).

As persistent chronic infection is one of the high-risk factors for malignant transformation of oral epithelial cells (Whitmore and Lamont, 2014), it means a lot to distinguish the distribution of oral flora between tumor patients and the normal population for tumorigenesis prevention. *P. gingivalis* is a key pathogen in periodontitis and the release of various virulence factors after infection may cause insufficient clearance of bacteria, which greatly increases the susceptibility to OSCC(Tezal et al., 2005; Hajishengallis, 2015; Lamont and Hajishengallis, 2015). The mechanism behind this is the bacterial component peptidoglycan (PDG) effectively activates the expression of RIPK2 and the downstream MAPK signal cascade, which ultimately upregulates programmed cell death-ligand 1 (PD-L1) to protect tumors from immune surveillance and clearance (Hirai et al., 2017; Groeger et al., 2020). It can be seen that NOD2/RIPK2 signaling pathway plays multiple roles in OSCC development, on one side, promoting the release of inflammatory factors to maintain the chronic inflammatory microenvironment; on the other side, boosting the expression of PD-L1 through MAPK signaling activation to help immune escape, both of which jointly promote the malignant progression of OSCC.

##### Glioma

The degree of tumor malignancy is closely related to the dysfunction of intracellular signaling pathways, among which the role of NF-κB and MAPK pathways in the tumorigenesis and progression of glioma has received more attention (Zhao et al., 2021). Studies have shown that the NF-κB pathway is constitutively activated and upregulated in glioma cells in response to different stimuli, since a negative feedback loop exists in the regulation of NF-κB, in which the key intermediate protein that determines the activation degree of NF-κB was found to be TNF receptor associated factor 3 (TRAF3) and the upstream regulator is RIPK2. On the one hand, RIPK2 negatively regulates the expression of TRAF3 to release the confinement to NF-κB; on the other hand, upregulated TRAF3 can in turn restrain RIPK2 expression (Cai et al., 2018). It is the imbalance of this negative feedback circle that leads to the malignant progression of glioma, which undoubtedly highlights the critical role of RIPK2-mediated NF-κB hyperactivation in pathogenesis.

Despite some current advances in multimodal treatment have been achieved, glioma is still one of the common tumors that seriously threaten human health (Sathornsumetee and Rich, 2006). Temozolomide (TMZ) is the first-line chemotherapy agent for the treatment of malignant glioma (Kaina, 2019). However, drug resistance to TMZ mainly contributes to unfavorable prognosis (Stupp et al., 2014; Hernandez-Duran et al., 2015). To figure out the exact mechanism of TMZ resistance, it is necessary to find key genes mediating drug resistance based on the distinction of gene expression patterns between resistant and sensitive tumors. Among the differentially expressed genes, RIPK2 presents higher expression in TMZ-resistant glioma than in sensitive glioma. Moreover, exogenous overexpression of RIPK2 induces activation of the NF-κB pathway and enhanced expression of O-6-methylguanine-DNA methyltransferase (MGMT), which is one of the downstreams of NF-κB. Both the activation of NF-κB and the upregulation of MGMT contribute to the reduced sensitivity to TMZ (Chen et al., 2018b; Hu et al., 2021). These results suggest that combined treatment with RIPK2/NF-κB/MGMT signaling pathway inhibitors and TMZ enhances the therapeutic efficacy in RIPK2-positive TMZ-resistant glioma.

**FIGURE 1:**
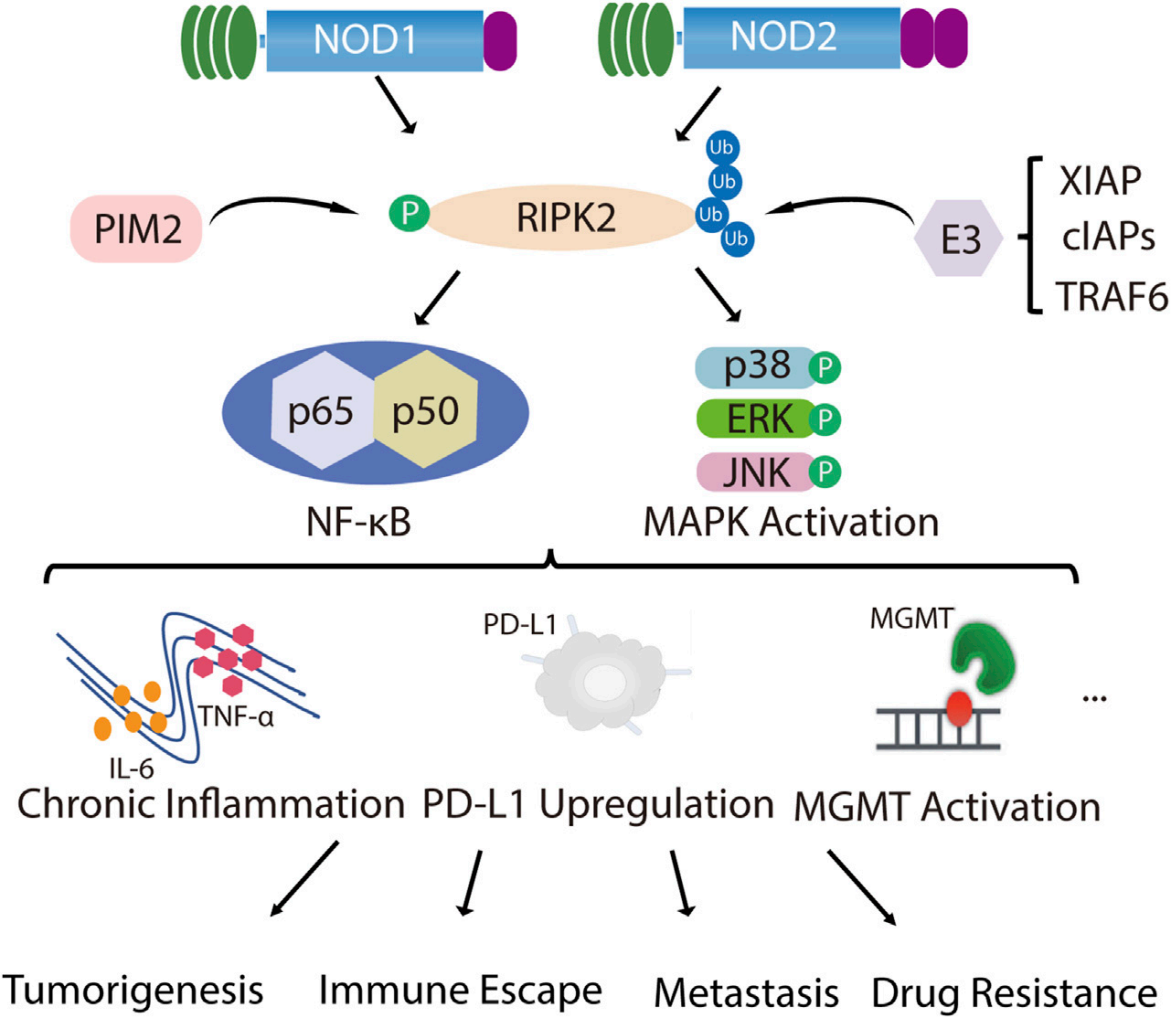
RIPK2 is closely related to tumor malignant progression. NOD1 and NOD2 recruit their common adapter RIPK2 through CARD-CARD interaction and then induce the phosphorylation of RIPK2, which further promotes ubiquitination of RIPK2 upon binding through TNF receptor associated factor (TRAF) family member TRAF6, inhibitor of apoptosis (IAP) family member XIAP and cellular inhibitor of apoptosis proteins (cIAPs), thereby recruiting and phosphorylating TAK1, TAB1 and TAB2/3, which ultimately induces MAPK (p38, ERK and JNK) and NF-κB activation and initiates downstream signaling cascades. In addition to NOD1/2, PIM2 also phosphorylates RIPK2, which induces NF-κB signaling pathway activation and promotes ERK phosphorylation. With the over-activated of MAPK and NF-κB signal, chronic inflammatory infiltration is formed, together with the up-regulation of PD-L1 or MGMT expression, mediating malignant proliferation, invasive metastasis, immune escape and drug resistance of tumors.

As a whole, in tumor types with high RIPK2 expression, RIPK2 can indeed effectively promote the malignant progression of the tumor, which has an undesirable effect on prognosis. The source of unfavorable impact mainly lies in the overactivation of the NF-κB signaling pathway and the changes in the immune microenvironment caused by a boost in pro-inflammatory factor secretion (Figure 1). Therefore, the effectiveness of RIPK2 as an anti-tumor target has been comprehensively verified, and the specific-targeting feasibility of RIPK2 will be further discussed in the following part.

## Development on RIPK2 inhibitors has achieved initial success

### Type I RIPK2 inhibitors that interact exclusively within the ATP-binding pocket

#### Gefitinib (Iressa) and erlotinib (Tarceva)

With tyrosine kinase activity, RIPK2 can undergo tyrosine autophosphorylation in response to NOD2 activation (Nembrini et al., 2009). In a small-scale screening, two epidermal growth factor receptor (EGFR) inhibitors—gefitinib and erlotinib—were found to exert an inhibitory impact on the tyrosine kinase activity of RIPK2, following the suppression of NOD2-induced NF-κB activation and cytokine release in NOD2 over-activation status. To determine whether these EGFR inhibitors act on RIPK2 directly, a RIPK2 mutant containing the homologous desensitizing mutation in the ATP-binding pocket (T95M) was generated and presented decreased sensitivity to the EGFR inhibitors. Coupled with the minor effect of erlotinib or gefitinib on lipopolysaccharide (LPS) or TNF signaling pathways, the direct inhibitory effect of erlotinib or gefitinib on RIPK2 was substantiated (Tigno-Aranjuez et al., 2010). However, the type I inhibitor gefitinib presented much lower activity in cells relative to *in vitro* assays because of its ATP-competitive action mode, which may be inactivated due to the high cellular concentration of ATP (Canning et al., 2015). Moreover, the lack of specificity set a limit to their application in RIPK2-related indications.

#### WEHI-345

WEHI-345 was identified through screening on a proprietary library containing 120 kinase inhibitors. This compound is an ATP analog with an IC_50_ value of 130 nM for RIPK2 kinase activity inhibition *in vitro*, which also exhibits superior selectivity. WEHI-345 functions mainly by binding to the ATP-binding pocket of RIPK2 and changing its conformation to inhibit NOD signaling, so that RIPK2 no longer binds to IAPs, for which RIPK2 ubiquitination and downstream NF-κB signaling activation interfere in succession. In the mice model, targeting RIPK2 with WEHI-345 was beneficial in nearly 50% of multiple sclerosis (MS) prevention (Nachbur et al., 2015). Virtually, the compound is currently only used as a tool drug, mostly owing to WEHI-345’s effect on NF-κB signaling is only to delay activation rather than block it completely. The exact reason is still pretty flimsy whether it is due to incomplete inhibition of RIPK2 kinase activity by WEHI-345 or an alternative RIPK2-independent pathway to activate NF-κB.

#### GSK583

By focusing the structure-activity relationship (SAR) strategy on the synergistic optimization of RIPK2 kinase potency and extensive kinome selectivity, GSK583, a RIPK2 candidate inhibitor, is stand out. The superior activity and high selectivity of GSK583 are explicitly proved by kinase activity, cytokine secretion. and organoid model verification. First, the IC_50_ of *in vitro* binding is 5 nM. In addition to its strong selectivity for p38α and vascular endothelial growth factor receptor 2 (VEGFR2), GSK583 shows excellent selectivity in the 300-kinase group at the concentration of 1 μM. At the cellular level, the IC_50_ values of TNF-α and IL-8 secretion by GSK583 are measured as 18 nM (human monocytes) and 8 nM (HEK), respectively. Following 1 μM GSK treatment, only an inhibitory effect on activated NOD1/2 signal is observed, whereas Toll-like receptors (TLR2/4/7) or cytokine receptors (like IL-1R) activation is rarely weak. Ultimately, an *ex vivo* culture system is used to evaluate the effect of GSK583 on spontaneous proinflammatory cytokine release in intestinal explants. It has been found that the production of TNF-α and IL-6 is significantly inhibited in a concentration-dependent manner in Crohn’s disease (CD) and ulcerative colitis (UC) samples, and the magnitude of inhibition is comparable to that of the steroid prednisolone. Despite its excellent kinase selectivity, GSK583 is the substrate of human Ether-a-go-go related gene (hERG) channels and CYP3A4, which hinders its further development as a drug candidate (Haile et al., 2016).

#### GSK2983559

The concerted activity at hERG ion channels and poor pharmacokinetics/pharmacodynamics (PK/PD) profile have imposed restrictions on the further progress of GSK583. To optimize for these flaws, the modulation of lipophilicity and strengthening of hinge binding capacity are conducted. These efforts first led to inhibitor **7**, which inhibited RIPK2 with higher potency, ameliorated human whole blood (hWB) activity, and reduced hERG activity (14 μM) (Haile et al., 2018; Haile et al., 2020). Further studies bring out the discovery of GSK2983559, which maintains hWB activity while not affecting the hERG channel. Although the solubility is imperfect, the design of phosphate ester prodrug provides more desirable pharmacokinetic properties across species as well as favorable activity in murine IBD models and UC/CD explants. Of note, GSK2983559 is the first RIPK2 inhibitor that has entered Phase I clinical trials (Haile et al., 2019). The single-center, randomized, double-blind and placebo-controlled phase I study was aimed to evaluate the safety, tolerability, pharmacokinetics, and pharmacodynamics of GSK2983559 in single (in both fed and fasted states) and repeated oral doses in healthy participants. Unfortunately, after the end of the phase I trial in 2019, GSK decided to terminate the further development of GSK2983559 due to non-clinical toxicology findings and reduced safety margins (data source: https://www.pharmcube.com/).

#### GSK-derived compounds

The Novartis RIPK2 inhibitor is obtained similarly to GSK583. After structural optimization, compound 8 can inhibit RIPK2 kinase activity at 3 nM. Even though compound 8 can selectively restrain muramyl dipeptide (MDP)-promoted cytokine production in both human peripheral blood mononuclear cells (hPBMC) and bone marrow-derived macrophages (BMDM), *in vivo* applicability plus kinome analysis have yet not been analyzed. Without these essential proving experiments carried out, and thus, it is equivocal whether the significant off-target effects exist or not (He et al., 2017). Different from compound 8, derivative 17 is obtained from GSK2983559 through cyclization strategy and structural optimization. 17 presents high affinity with RIPK2 (Kd = 5.9 nM) and high degree of discriminability towards receptor-interacting protein kinase 1 (RIPK1, Kd > 30,000 nM). Besides *in vivo* effectiveness, 17 displays good metabolic stability and no cytochrome P450 (CYP) inhibition (Wu et al., 2022).

To discover structurally diverse inhibitors of RIPK2, the fragment-based drug design (FBDD) procedure begins by screening the GSK compound collection. Subsequently, by employing the principles of fragment evolution and robust crystallography, pyrazolocarboxamide 11 is found a potent and selective ATP-competitive RIPK2 kinase Inhibitor based on structure-based design. Even though the IC_50_ of 11 is 30 nM and compared with activin receptor-like kinase 5 (ALK5), VEGFR2, and lymphocyte-specific protein tyrosine kinase (LCK), it shows more than a hundredfold inhibitory activity, the selectivity for same-family proteins is still unknown (Haffner et al., 2019).

#### BI 706039

As a potent and specific functional inhibitor of RIPK2, BI 706039 effectively blocks MDP-induced TNF-α production from human (IC_50_ < 1.0 nM) and mouse cells (IC_50_ = 2.9 nM). Besides, it has a more than 500-fold selectivity on other pattern recognition receptor pathways as well as favorable pharmacokinetic properties. To further analyze the *in vivo* effectiveness of BI 706039, the T-bet/Rag2 double knockout (TRUC) mouse model of IBD is introduced to evaluate the potency of BI 706039 on intestinal inflammation. Oral, daily administration of BI 706039 with distinct dosages all displays improvement in colonic histopathological inflammation, colon weight, and protein terminal levels normalized for fecal lipocalin, the greatest remission appears at the dose of 2.5 mg/kg. Taken together, it is suggested that a relatively low dose of BI 706039 can lead to a significant improvement in intestinal inflammation (Ermann et al., 2021).

#### OD36 and OD38

With the development of a proprietary novel small molecule macrocyclization platform—Oncodesign, lead compound optimization has got iterative improvement until the desired selectivity and metabolic half-life properties are achieved. Early RIPK2-specific compounds (OD 36 and OD38) screened by this technique have shown ideal inhibitory activity against RIPK2 both *in vitro* and *in vivo* using an MDP-induced peritonitis mice model. Both OD36 and OD38 show high potency with IC_50_ values in the lower nanomolar range (5.3 nM and 14.1 nM for OD36 and OD38, respectively). However, while maintaining strong inhibitory activity against RIPK2, the off-target effects increased at higher concentrations with OD36 given. Things are different when it comes to OD38, whose inhibitory effect is rarely influenced by concentration alteration (Tigno-Aranjuez et al., 2014). These results are useful in ascribing alleviating effects of IBD symptoms directly to RIPK2 inhibition.

#### Activin receptor-like kinase-2 (ALK2) inhibitor derived RIPK2 inhibitors

It has been reported in the literature that various ALK2 inhibitors oftentimes demonstrate inhibitory activity against RIPK2(Mohedas et al., 2013; Mohedas et al., 2014). Encouraged by this correlation, SAR analysis has been conducted to find new structures. A new series of RIPK2 kinase/NOD signaling inhibitors based on a 3,5-diphenyl-2-aminopyridine scaffold was developed. Representative compounds are numbered CSLP37 and CSLP58. The IC_50_ value of CSLP37 on RIPK2 is 16 ± 5 nM while on NOD cell signaling is 26 ± 4 nM. At the same time, CSLP37 shows more than 20-fold selectivity *versus* ALK2 (Suebsuwong et al., 2020). In order to further improve the selectivity for ALK2, another series with the core of pyrido [2,3-day]pyrimidin-7-one was designed. Compared with CSLP37, the representative compound UH15-15 inhibits the RIPK2 kinase with IC_50_ of 8 ± 4 nM and presents more than 300-fold selectivity compared to ALK2. Additionally, UH15-15 has *in vitro* absorption, distribution, metabolism, excretion (ADME) and pharmacokinetic characteristics, which further support the feasibility of applying UH15-15 as a new RIPK2 inhibitor (Nikhar et al., 2021).

#### RIPK2 inhibitors with novel structures

Through structure-based drug design, RIPK2 inhibitors with new cores derived from the Fms related receptor tyrosine kinase 3 (FLT3) inhibitor CHMFL-FLT3-165 are discovered. Among the series, compound 10w is identified as a particularly potent RIPK2 inhibitor with an IC_50_ of 0.6 nM, which shows the sub-nanomolar RIPK2 inhibitory activity. In the mice model of acute colitis, 10w exerts a better therapeutic effect than the WEHI-345 and Janus kinase (JAK) inhibitor fegotinib or tovaxicin, as demonstrated by weight loss, tissue inflammation, and disease activity index (DAI) score. A study on this compound is still in its infancy, the pharmacokinetic properties still need to be further optimized (Yuan et al., 2022). In addition, a patent (WO 2020/132384 A1) demonstrated a series of thienopyridines as novel RIPK2-specific inhibitors. The results from standard time-resolved fluorescence energy transfer (TR-FRET) screening assay for RIPK2 inhibition revealed that compounds 1, 40, 46, 75, 105, and 120 among the series all display commendable inhibitory activity with IC_50_ less than 10 nM (Sabnis, 2020).

### Type II RIPK2 inhibitors that target the inactive “DFG-out” conformation of the kinase domain

#### Ponatinib

Type II inhibitors also provide a new tool for kinase inhibition. As the first to be found, ponatinib blocks RIPK2 kinase activation without affecting the C-terminal CARD domain and its engagement with NOD2. Phosphor-activated RIPK2 is subsequently polyubiquitinated by multiple E3 ligases, ponatinib can completely inhibit this modification, whereas type I inhibitors previously only caused a postponement in ubiquitination. Meanwhile, ponatinib displays excellent selectivity toward MDP-dependent signaling relative to LPS-dependent pathways (Canning et al., 2015). But ponatinib is also a potent inhibitor of RIPK1 and receptor-interacting protein kinase 3 (RIPK3) kinase activity, making it a pan-RIPK inhibitor (Najjar et al., 2015).

#### CSR35

The type II pan-kinase inhibitors ponatinib and regorafenib, with IC_50_ values of 7 and 41 nM, respectively, could serve as initial templates for RIPK2 activation loop targeting (Canning et al., 2015). In order to improve the selectivity, another design of RIPK2 inhibitors based on pan-kinase inhibitor regorafenib has appeared, with the purpose of engaging basic activation loop residues Lys169 or Arg171. The proof-of-concept of the design strategy for RIPK2 activation loops is achieved by introducing the carboxylic acid fragment into regafenib. And a series of CSR products, among which CSR35 is the best, is obtained. X-ray crystallography confirms the interaction of CSR35 with RIPK2, including ion-ion contacts with Lys69 side chains, and IC_50_ values of CSR35 demonstrate modest percent inhibition in the initial assessment, to be specific, 2.26 ± 0.11 µM. In addition, derivatives lacking hinge binding groups that remain mild inhibitory activity can be regarded as scaffolds for designing inhibitors engaged with the activation loop, such as type III, which means, given the diversity of activation loop kinase segments, this strategy can bring forward additional design solution for improving the selectivity of similar inhibitors (Suebsuwong et al., 2018).

#### RIPK2 inhibitor 1

By employing similarity-based virtual screening and molecular docking analysis, RIPK2 inhibitor 1, which has a similar binding pattern to ponatinib, is identified. RIPK2 inhibitor 1 empirically blocks RIPK2 autophosphorylation as well as NF-κB signaling, which finally attenuates lung and intestinal inflammation at the dosage of 1–2 μg/g. Even though RIPK2 inhibitor 1 demonstrates *in vitro* inhibition of several other kinases, consisting of a 20%–30% inhibitory effect on c-ABL, Aurora B, or HER2, it may be due to the off-target effect indirectly aroused by resolving the inflammation (Salla et al., 2018).

### RIPK2 proteolysis-targeting chimeras (PROTACs)

**FIGURE 2:**
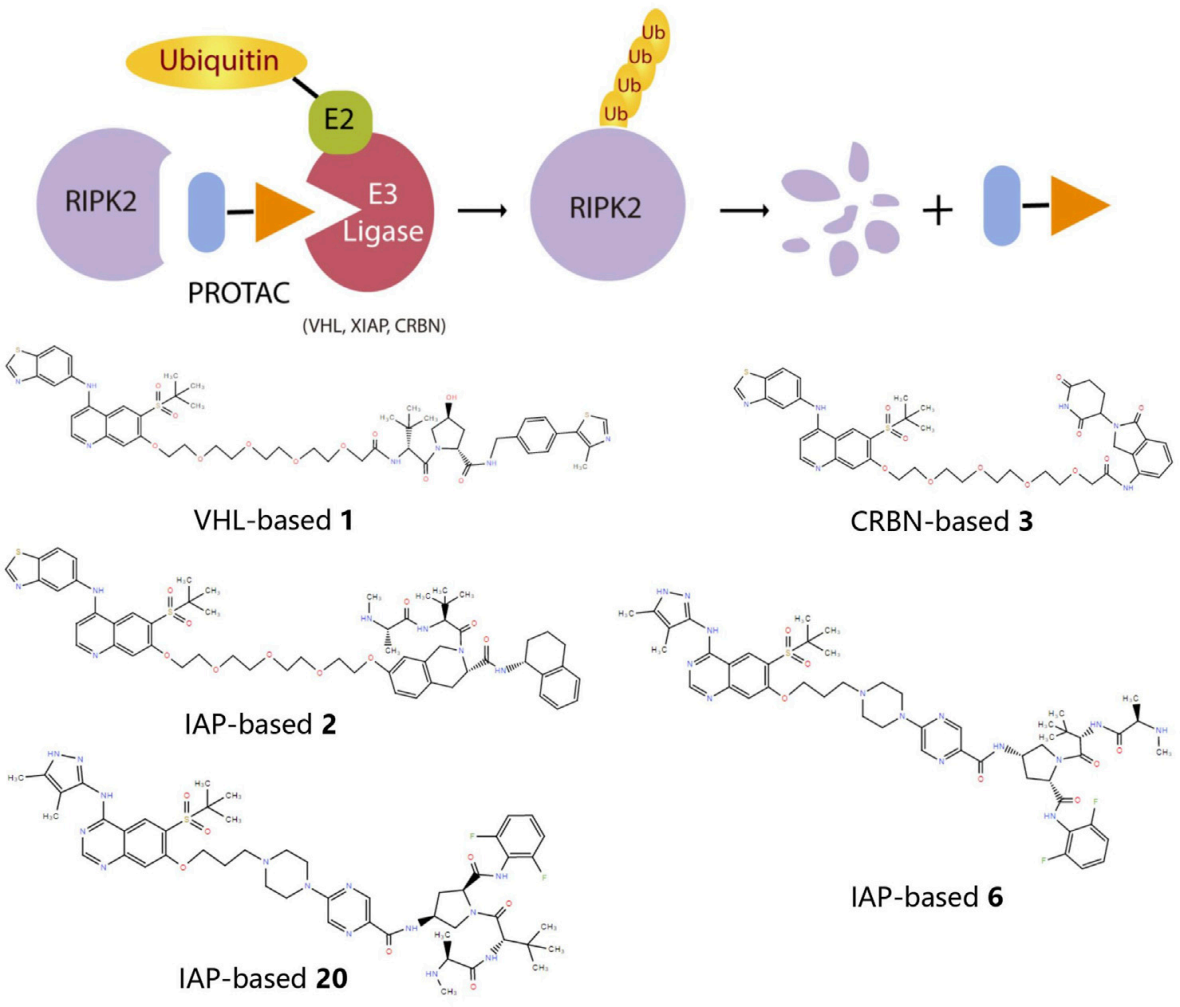
The action principle and structure of RIPK2 PROTACs. Protein turnover data for RIPK2 obtained by dynamic stable isotope labeling with amino acid in cell culture (SILAC) labeling experiments indicate that RIPK2 typically has a half-life of ∼50 hours or longer (Mathieson et al., 2018). The extended half-life makes RIPK2 a suitable candidate protein to explore the potential of PROTAC-mediated target degradation for prolonged PD responses.

Considering the half-life of RIPK2 protein is 50 h or longer (Doherty et al., 2009), the prolonged half-life makes RIPK2 a prospective candidate protein to explore the potential for extended PD response from PROTAC-mediated target degradation (Figure 2). The disclosed structures of potent and selective RIPK2 PROTACs include the Von Hippel-Lindau tumor suppressor (VHL)-based RIPK2 PROTAC 1 and analogous PROTACs 2 and 3, where the VHL binder is substituted with IAP and Cereblon (CRBN)-based E3 ligase recruiting moieties, respectively. All PROTACs mentioned above degraded RIPK2 in a concentration-dependent manner. The IAP-based PROTAC 2 is found to have a pDC_50_ value of 9.4 ± 0.1, which presents better degradation efficacity than VHL-based PROTAC 1 (pDC_50_ 8.7 ± 0.1) and CRBN-based PROTAC 3 (pDC_50_ 8.6 ± 0.4) (Bondeson et al., 2015; Mares et al., 2020).

PROTAC 6 optimized according to 2 produces the concentration- and time-dependent decrease in RIPK2 protein level in human PBMCs, with a pDC_50_ of 9.4 ± 0.2. At the same time, 6 significantly increases the binding capacity to RIPK2, thereby acquiring an extremely selective binding profile and low off-target probability. Specifically, RIPK2 is the only detected degradation target of PROTAC 6 at a concentration below 0.1 µM. The PK/PD study also reveals that repeated administration results in the cumulative impact on protein degradation of RIPK2 without drug accumulation. Considering the relatively slower rate of protein synthesis, dosing at longer intervals can still provide sustained efficacy (Mares et al., 2020). Further optimization focuses on improved solubility and increased human/rat microsomal stability, and PROTAC 20 possesses the best overall profile with good solubility, effective degradation of RIPK2, and accompanying inhibition of inflammatory cytokine release. Moreover, the utilization of a slow-release matrix makes the long-acting parenteral formulation last longer than 1 month feasible (Miah et al., 2021).

Overall, most reported RIPK2 inhibitors have been ATP-competitive type I molecules, among which mainly originate from the EGFR inhibitor gefitinib (Tigno-Aranjuez et al., 2010) (Table 1). However, there are relatively few type II inhibitors, which are mainly designed and modified based on the binding mode of ponatinib and RIPK2. More importantly, due to the higher affinity between endogenous full-length RIPK2 and ATP and the preferred DFG-out conformation, the *in vitro* kinase potency and cellular activity of ponatinib vastly outperforms the type I inhibitor (Canning et al., 2015). Nonetheless, the optimized type II inhibitors cannot show comparable effects, even worse. The most likely explanation is that the existence of a hinge-binding ‘‘head” is as important as the allosteric pocket, that is to say, the kinase inhibitor with both type I and type II characteristics is the best choice.

**TABLE 1:** Structures and Inhibitory Activity of Type I and II RIPK2 Specific Inhibitors

Name	Structure	Type	Kinase IC_50_	Reference
WEHI-345	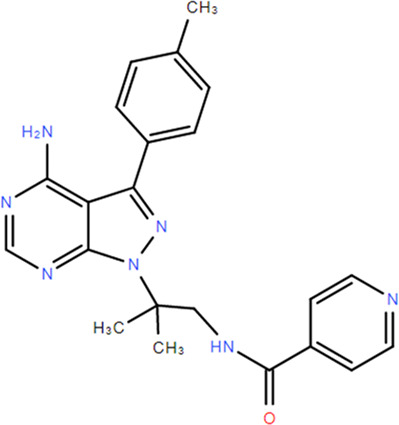	I	130 nM	Nachbur et al. (2015)
GSK583	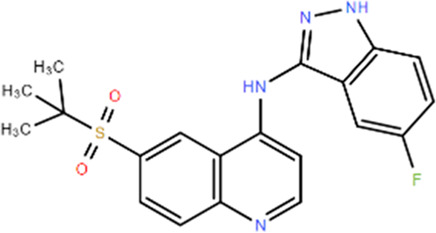	I	5 nM	Haile et al. (2016)
GSK2983559	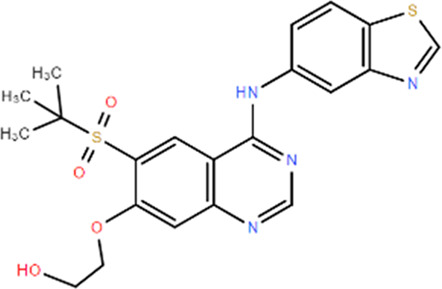	I	2 nM	Haile et al. (2019)
Compound 8	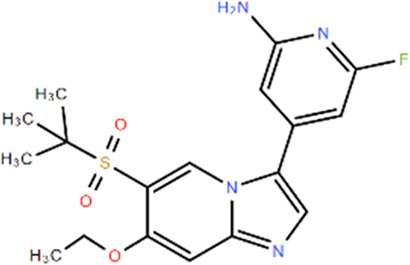	I	3 nM	He et al. (2017)
Derivative 17	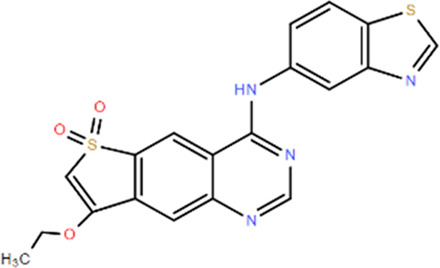	I	Unknown	Wu et al. (2022)
Pyrazolocarboxamide 11	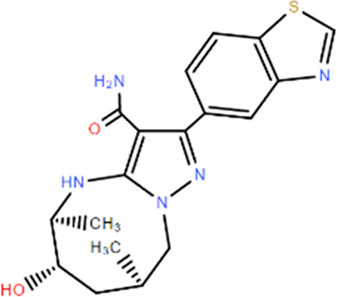	I	30 nM	Haffner et al. (2019)
OD36	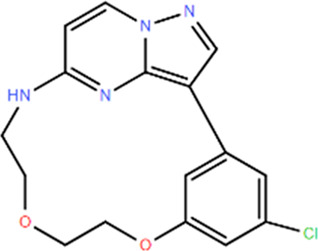	I	5.3 nM	Tigno-Aranjuez et al. (2014)
OD38	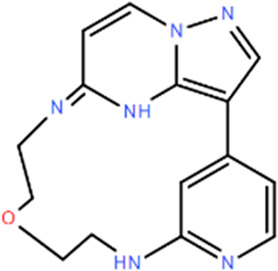	I	14.1 nM	Tigno-Aranjuez et al. (2014)
CSLP37	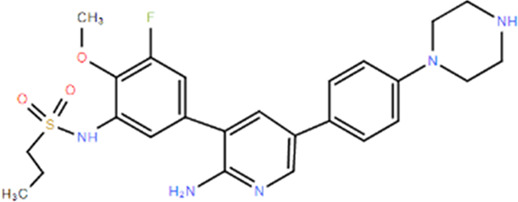	I	16±5 nM	Suebsuwong et al. (2020)
UH15-15	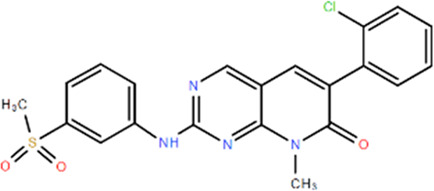	I	8±4 nM	Nikhar et al. (2021)
Compound 10w	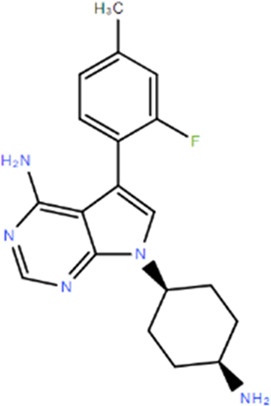	I	0.6 nM	Yuan et al. (2022)
CSR35	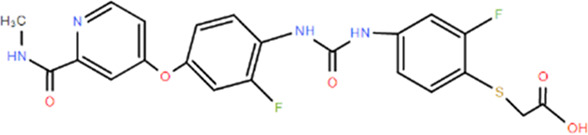	II	2.26±0.11 µM	Suebsuwong et al. (2018)
RIPK2 inhibitor 1	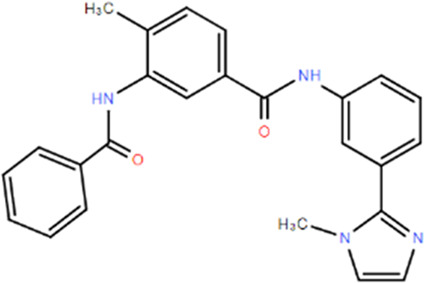	II	Unknown	Salla et al. (2018)

As for anti-tumor therapy, we deem that the application of RIPK2 kinase inhibitors is superior to RIPK2 PROTACs. Firstly, most tumors are in the NF-κB hyperactivation status, and as the direct upstream of NF-KB, the functional inhibition of RIPK2 can better control the activation degree of the NF-κB signaling pathway. Secondly, RIPK2 expression level has great heterogeneity in tumors, and the correlation between RIPK2 expression level and prognosis in different types of tumors can be opposite. Third, RIPK2 still plays a normal function in innate immunity, excessive degradation of RIPK2 protein may destroy its original physiological function, which means that the therapeutic window of PROTACs will be narrow.

## Perspective

It is generally believed that inflammation, especially chronic inflammation, is closely connected with tumor proliferation, angiogenesis, and immune escape (Korniluk et al., 2017; Greten and Grivennikov, 2019; Zhao et al., 2021). As one of the representatives of inflammatory immune receptors, NOD-like receptors mainly participate in the innate inflammatory immune response by mediating the NF-κB, MAPK, and autophagy-related pathways (Motta et al., 2015). However, the role of NOD1/2 in cancer is complex. More precisely, NOD1 exerts its anti-tumor effects through the induction of apoptosis, but uncontrolled apoptosis mediated by NOD1 might induce immunosuppression microenvironment, thereby promoting tumor progression. Furthermore, the anti-inflammatory function of NOD2 is dependent on the recognition and binding of bacteria and their derivatives, and when this association is inadequate, NOD2 contributes to chronic inflammation and promotes cancer (Zhang et al., 2022b; Wang, 2022). Hence, direct targeting NOD1/2 against tumor may result in a therapeutic effect that contradicts expectations.

Unlike NOD1/2 inhibitors, targeting the downstream protein of NOD1/2 signaling pathway, RIPK2, to fight against cancer is more advantageous for the following three reasons. Firstly, RIPK2 is highly expressed in a variety of tumors, especially in breast and colon tumors (Jaafar et al., 2018; Jaafar et al., 2021); secondly, the molecular mechanisms that RIPK2 promotes tumor progression are multi-faceted, containing both mechanisms related to the regulation of the tumor immune microenvironment and mechanisms related to oncogene amplification or transcription factor addiction that are independent to its primal immune regulatory function, which means RIPK2 inhibition is more destructive to tumors; finally, the development of RIPK2 inhibitors is currently the most advanced compared with other key proteins in the NOD1/2 signaling pathway, even if the only clinical trials have temporarily ended in failure, through continuous optimization of compound structures and updating of inhibitor design inspirations based on the results of the existing preclinical tests, we believe that there will be molecules with better activity and safety.

Regarding the application of RIPK2 inhibitors in tumor therapy, we hold the opinion that three main aspects could be considered. First, is prescribing the RIPK2 inhibitor alone. It has been reported that the small molecule cRIPGBM selectively induces apoptosis in glioblastoma multiforme cancer stem cells (GBM CSCs) *in vitro* and significantly decrease tumor size in the xenograft mouse model. Mechanistically, cRIPGBM directly interacts with RIPK2, which results in decreased association with TAK1 and increased association with caspase 1, leading to downstream activation of the apoptotic signaling cascade. Given the high rate of GBM tumor relapse and therapeutic resistance, the observed sensitivity of GBM CSCs to RIPK2-induced apoptosis has profound implications for the development of new therapies for GBM (Lucki et al., 2019).

Second, RIPK2 inhibitors can be used in combination with chemotherapeutic agents. The PIM2-upregulated phosphorylation level of RIPK2 enhances HCC cells’ ability to tolerate 5-Fluorouracil (5-FU) and cisplatin (Tang et al., 2020). In GBM treatment, silencing of RIPK2 enhanced cellular sensitivity to TMZ, which offers a novel strategy for RIPK2-positive TMZ-resistant glioma (Hu et al., 2021). Moreover, DAMPs produced during paclitaxel treatment can activate the NOD2 signaling and worsen the tumor microenvironment, resisting the therapeutic effect of paclitaxel, but NOD2 antagonist enables the sensitization of chemotherapeutic response (Dong et al., 2017). As a part of the NOD2 signaling pathway, RIPK2 inhibition may offer the equivalent therapeutic benefits.

Last, is the attempt of coupling RIPK2 inhibitors with immune checkpoint inhibitors (ICIs) in cancer treatment. Inflammatory breast cancer (IBC) is type of tumors that remains a significant challenge. As it is often poorly responsive to conventional therapies, investigators have been eager to determine whether ICIs will bring benefits to IBC patients. According to a recent Phase 2 trial, single-agent treatment with anti-programmed cell death 1 (PD-1) antibody pembrolizumab as maintenance therapy for metastatic IBC has reported a disease control rate of 47% after 5 months (Gao et al., 2020). To further improve the response rate, RIPK2 inhibitors can be introduced for the reason that the associated “molecular inflammation” is the driving force behind IBC tumorigenesis and metastasis (Zare et al., 2018). The upregulated expression of RIPK2 and activation of inflammatory mediators, especially the hyperactivated stage of NF-κB signaling in IBC makes it more reasonable for synergy between RIPK2 inhibitors and ICIs.

Admittedly, what we cannot be ignored is the fact that NODs and their downstream RIPK2 can not only promote the proliferation, metastasis, and invasion of tumors, but also exert an inhibitory effect on tumor progression as immune responders, which may be related to different tumor types on the one hand. On the other hand, NODs and RIPK2 in the early stage can activate the adaptive immune response, thereby killing tumor cells; however, overactivated such proteins may be the prime mover behind aggravated inflammation and tumor progression in the advanced stage. This means that precise differentiation and staging of tumors with the indicated phenotype, like excessive activation of NF-κB, has important reference value for the application of RIPK2 inhibitors as anti-tumor drugs or biological modulators enhancing the effectiveness of antineoplastic agents.
